# Using Intervention Mapping to develop a programme to prevent sexually transmittable infections, including HIV, among heterosexual migrant men

**DOI:** 10.1186/1471-2458-7-141

**Published:** 2007-07-05

**Authors:** Mireille EG Wolfers, Caty van den Hoek, Johannes Brug, Onno de Zwart

**Affiliations:** 1 Municipal Public Health Service Rotterdam Area, Rotterdam, the Netherlands; 2 Department of Public Health, Erasmus MC, University Medical Centre Rotterdam, Rotterdam, the Netherlands

## Abstract

**Background:**

There is little experience with carefully developed interventions in the HIV/STI prevention field aimed at adult heterosexual target groups in the Netherlands. The ability to apply intervention development protocols, like Intervention Mapping, in daily practice outside of academia, is a matter of concern. An urgent need also exists for interventions aimed at the prevention of STI in migrant populations in the Netherlands. This article describes the theory and evidence based development of HIV/STI prevention interventions by the Municipal Public Health Service Rotterdam Area (MPHS), the Netherlands, for heterosexual migrant men with Surinamese, Dutch-Caribbean, Cape Verdean, Turkish and Moroccan backgrounds.

**Methods:**

First a needs assessment was carried out. Then, a literature review was done, key figures were interviewed and seven group discussions were held. Subsequently, the results were translated into specific objectives ("change objectives") and used in intervention development for two subgroups: men with an Afro-Caribbean background and unmarried men with a Turkish and Moroccan background. A matrix of change objectives was made for each subgroup and suitable theoretical methods and practical strategies were selected. Culturally-tailored interventions were designed and were pre-tested among the target groups.

**Results:**

This development process resulted in two interventions for specific subgroups that were appreciated by both the target groups and the migrant prevention workers. The project took place in collaboration with a university center, which provided an opportunity to get expert advice at every step of the Intervention Mapping process. At relevant points of the development process, migrant health educators and target group members provided advice and feedback on the draft intervention materials.

**Conclusion:**

This intervention development project indicates that careful well-informed intervention development using Intervention Mapping is feasible in the daily practice of the MPHS, provided that sufficient time and expertise on this approach is available. Further research should test the effectiveness of these interventions.

## Background

The number of registered new sexually transmitted infections (STI) in the Netherlands has been increasing for several years [1], and reinforcement and extension of preventive actions is necessary. In the Netherlands, as well as in other Western European countries, ethnic minority groups originating from countries with a high prevalence of heterosexually transmitted HIV (Sub-Saharan Africa, Caribbean region) have higher HIV incidence levels. These groups are therefore recognized as important target populations for prevention [1-4]. Heterosexual migrant men are a priority group, in particular because little specific efforts have been made to improve their sexual health [5], and because of the contribution of men to the spread of STI.

Since the 1990s, it has been recognized that prevention interventions should be developed systematically on the basis of evidence and theory [6], because such a planned procedure substantially improves the chance of success [7-10]. Recently the Intervention Mapping (IM) protocol [11] was introduced as a more elaborate tool to guide each step in the process of intervention development and implementation. Interventions carefully developed on the basis of theory and evidence have been documented for men having sex with men, students and drug users [12-19]. However, evidence based interventions aimed at ethnic minority groups in HIV and STI prevention are scarce in both the Netherlands and abroad. In the HIV prevention field the ability to apply IM in daily practice, outside of academia is a matter of concern [20]. The present paper describes the theory and evidence-based development of HIV/STI prevention interventions for two groups of heterosexual migrant men, those with an Afro-Caribbean (AC) and those with a Turkish/Moroccan (TM) background, organised by the Municipal Public Health Service (MPHS) of Rotterdam Area. The project was conducted by a team of two prevention workers, a researcher in health education and health promotion and a health policy adviser experienced in IM, in frequent consultation with a university-based expert in planned health promotion.

## Methods

### Target population

The Rotterdam area is typical of many major urban centres in Western Europe, in that a large proportion (46% in 2005) of the population has a non-native ethnic background [21]. The largest ethnic minority groups in Rotterdam comprise those born in, or whose parents came from, Surinam, Dutch-Caribbean Islands, Cape Verde, Morocco and Turkey (these groups: approx. 170,000 people). Since we could not address each group within the present project, we decided to develop interventions for two specific sub-populations: men with an AC background (Dutch-Caribbean, Surinam or Cape Verde) and men with a TM background.

### Intervention Mapping

**Figure 1:**
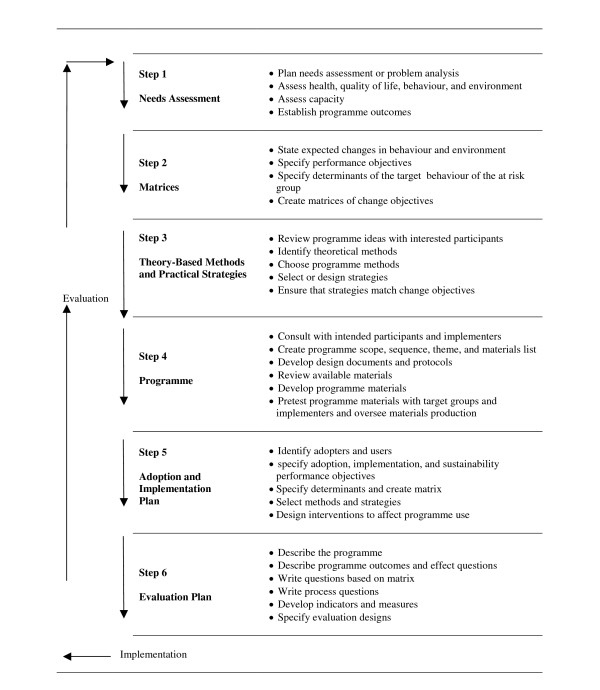
Intervention Mapping protocol [11].

IM is a stepwise approach for theory and evidence based development and implementation of interventions. It comprises six steps; each leading to a product that guides the next step (see Figure 1) [11]. The first step is to conduct a needs assessment. Interventions should be developed for health problems that are serious and prevalent enough for the allocation of scarce resources. Subsequently, the behavioural risk factors that contribute to these problems should be identified. In order to guide the search for modifiable factors, individual and environmental determinants of the risk behaviour are investigated too. These investigations involve application of behavioural determinant theories such as the Theory of Planned Behaviour [22], Social Cognitive Theory [23], Protection Motivation Theory [24], and of social ecological models [25] which address potential personal behavioural determinants like attitude, self-efficacy, social norms, risk perception, as well as social- cultural and environmental factors [26]. The needs assessment ends with defining the most distant objectives in the IM model: desired (behavioural) *outcomes*. These outcomes should be formulated in terms of desired reductions in the health problems at stake, or in terms of desired behavioural and/or environmental changes that are linked to the health problems identified in the needs assessment. For the present project, the following *behavioural change *was defined as outcome: *'consistent condom use among heterosexual migrant men for the prevention of HIV and STI with new and casual partners'*.

In the second step of IM a further specification of the objectives is made. First the behavioural outcomes are delineated into component parts, i.e. specific actions that should lead to the desired behaviour, the so-called *performance objectives *(PO; what the target group members need to do as a result of the programme, see Table 2). Next, the most important and changeable behavioural determinants of these performances should be identified and selected. Again, explanatory behaviour theories are used to identify determinants that are strongly associated with the risk behaviour. Change theories and theoretical change methods are then used to assess the changeability of the determinants. Behavioural change theories focus on ways to change behaviour. Examples of such theories are the Transtheoretical model and its stages of change construct [27], the Precaution Adoption Process Model [28], Goal Setting Theory [29], and Social Cognitive Theory [23]. Finally, *change objectives *are formulated; those are the most immediate targets of a program to be accomplished and are stated in terms of what a person exactly needs to learn to enable performance of the specific behaviour changes. Combining the PO with its most important and changeable determinants results in matrices that helps in the identification of possible interventions. In the present project, an example of a change objective is that a man describes where he puts away his condoms in order to always have them available (a preparation), with as associated PO that a man always carries his condoms (Table 3).

**Table 2:** Performance objectives by target group

**Turkish and Moroccan unmarried men**	**Afro-Caribbean men**
Man decides to use a condom at every sexual contact.	Man decides to use a condom with new and casual partners.
Man buys condoms.	Man distinguishes realistically between casual and steady partners.
Man carries condoms always.	Man buys condoms.
Man brings out a condom at relevant moment.	Man always carries condoms.
Man uses condoms correctly.	Man brings out a condom at relevant moment.
Man uses condoms correctly and consequently in every sexual contact.	Man uses condoms correctly.
	Man uses condoms correctly and consequently with new and casual partners.

**Table 3:** Matrix cells of change objectives for heterosexual migrant men:selected examples regarding self efficacy

Performance objectives	Change objectives
Man decides to use a condom at every sexual contact.	Man expresses confidence that he will use condoms, also in a situation that a woman does not request a condom.
Man buys condoms.	Man describes where he can buy condoms, also after shop closing time.
Man always carries condoms.	Man describes where he puts away his condoms, to have them available always. Man describes a plan how he carries condoms always.
Man brings out a condom at relevant moment.	Man describes a plan how he takes a condom out at the right moment.
Man uses condoms correctly.	Man demonstrates how he uses condoms correctly Man expresses confidence to use condoms correctly.
Man uses condoms correctly and consequently in every sexual contact.	Man expresses confidence to resist unsafe sex.

After this goal-setting process, IM step 3 is the selection of useful theory-based and evidence-based learning and behavioural change strategies that are applicable to the change objectives. Behavioural change theories are used when selecting and specifying intervention strategies and methods that should lead to the desired changes. In step 4 the intervention programme is then designed: the methods and strategies are translated into intervention materials. In IM steps 5 and 6 a detailed plan for the adoption and implementation and for evaluation is produced. The present paper focuses on steps 1–4.

#### Intervention Mapping step 1: needs assessment

The needs assessment was carried out in two steps: a literature review and additional qualitative research. First, a short epidemiological analysis on STI and HIV was done by reviewing literature and data from the Erasmus University Medical Centre in Rotterdam. Then available literature on behavioural risk factors and behavioural determinants was reviewed. Because studies on determinants of safe sex among our target group were hardly available, a qualitative study was conducted to explore these determinants within the present project. Initially, key figures of the communities (n = 12) and two nurse-counsellors working with HIV patients in the Erasmus Medical Centre were interviewed. Subsequently, seven focus group discussions were conducted with men of the five ethnic groups that make up the target population.

The focus group discussions were conducted according to a semi-structured interview protocol that was developed in collaboration with an expert-group. This protocol was derived from social cognitive theory [23] and from social-ecological models of health behaviour [25]. Therefore, behavioural determinants at the personal level (outcome expectations, attitudes, and self-efficacy) and at environmental levels were discussed. MPHS health educators from the same communities recruited the respondents for the group discussions from their networks and during regular health education activities. They also put announcements on poster boards at community centres. The discussions started after an informed verbal consent was obtained and lasted between 55 and 85 minutes. The men could participate anonymously; all participants received a small financial reward and a packet of condoms for their collaboration. The focus group discussions were tape-recorded, transcribed and thematically analysed. The Medical Ethical Review Board of Erasmus MC did not require formal approval for the study because this study is not subject to the Medical Research Involving Human Subjects Act (WMO).

The seven focus group discussions were conducted separately with AC men between 16 and 25 years old (n = 5, three men originating from Surinam, one from the Dutch-Caribbean, one from Cape Verde; average age 21), with AC men aged 25 or above (n = 3, two from Surinam and one from Cape Verde; average age 30), with two groups with married TM men (4 and 9 participants, respectively; average age 39), and with three groups of unmarried TM men (4, 6 and 7 participants, respectively; average age 18). Most participants in the groups of younger AC men and married TM men knew each other and decided to join the discussion together. The married TM men were recruited at a garage that serves as a meeting place, so some of them were friends. The older AC men were strangers to each other. The respondents were higher educated than average migrants in the Netherlands: two-thirds of them had a low education and one-third had a higher education. The young and unmarried men were born in the Netherlands or had migrated to the Netherlands in their early childhood. Of the older and married respondents, 75% migrated to the Netherlands at adult age.

#### Intervention Mapping step 2: matrix of change objectives

In order to decide on the specific desired behaviour change, we discussed the outcomes of the needs assessment in expert meetings with representatives of the migrant prevention field and experts on behaviour change and HIV/STI prevention. In this stage, the final PO and most important changeable behavioural determinants were identified. By combing these, a matrix of change objectives was constructed, which was discussed with a behavioural change expert.

#### Intervention Mapping step 3: selecting suitable theoretical methods and practical strategies

First an inventory of literature on methods and strategies was made. Then we held a brainstorm session with prevention officers and experts working in the field of STI/HIV prevention at the MPHS Rotterdam Area on successes that they experience in their daily activities. We also encouraged the prevention officers to think of intervention techniques they had used, for example to change specific determinants that were identified in step 2 (e.g. self-efficacy and skills to buy or use condoms). We examined the suggested intervention techniques in order to identify their underlying effective components and to judge whether these techniques could be used to address the change objectives from step 2.

Based on the brainstorm session and the literature inventory, a discussion session was held among the project group members. Our aim was to identify which intervention tools and activities were most suitable for addressing the selected change objectives. We had to take into account the given restraints in time and finances, as well as the characteristics of the target population and the abilities of the MPHS health educators delivering the interventions. This led to the choice of several ideas for practical intervention techniques derived from theory-based methods which were identified in literature and translated into practical ideas, aided by the experience of the prevention officers. Then the project group made a draft of the intervention components. This draft was discussed with an expert on behavioural change before the project group made final decisions on the intervention strategies and components.

#### Intervention Mapping step 4: designing a programme plan

The strategies chosen in step 3 were translated into plans for actual intervention components combined with a comprehensive practical intervention plan. In the phase of programme design the prevention officers played the most important role by designing materials in collaboration with the target groups. To this end, we organised two extra focus group discussions among TM and AC men to gather more information on specific potential determinants (i.e. specific outcome expectancies regarding condoms, and perceived availability and accessibility of condoms) and to discuss the preliminary ideas for the intervention. The prototypes were pilot-tested among members of the target groups and were adapted where necessary. The MPHS researcher gave theoretical input and guidance during this phase.

## Results

### Outcomes of the Intervention Mapping process

#### Step1: Needs assessment

##### Analysis of the health problem

STIs are the most common infectious diseases in the Netherlands, with 60,000 Chlamydia; 25,000 Genital warts and 12,000 Genital herpes infections yearly [30]. Some STIs, such as Chlamydia, can lead to severe health problems and may first occur without symptoms. HIV infection, despite improvements in treatment, is still an incurable disease. Among adults in Rotterdam, more than half of new HIV-diagnoses since 1999 and 61% in 2004 are among persons belonging to ethnic minority communities (Unpublished data, Erasmus Medical Centre, Rotterdam), while in 2004, 46% of the Rotterdam population belonged to a ethnic minority community [21]. Annual Dutch surveillance data show that a relatively high number of migrants is represented in new STI diagnoses [1]. The figures of 2004 show that males from Surinam and the Dutch-Caribbean accounted for 12% of all male Chlamydia diagnoses and 16% of the gonorrhoea cases [1], while these groups represent 2.8% of the Dutch population [31]. The national Chlamydia survey of 2002/2003 revealed a three times higher prevalence among Surinamese and Dutch-Caribbean men and a 4.5 times higher prevalence among Turkish and Moroccan men than among native Dutch men [32].

##### Analysis of behaviour

In studies among persons with a AC descent, a high prevalence of sexual risk behaviour among men was observed, compared to native Dutch men [33-37]. Recent studies in Rotterdam and Amsterdam indicate that men engaged in unsafe sexual behaviour more often than women and were more likely to use condoms inconsistently [33,34,37-39]. These men also reported more unsafe sexual behaviour in the past five years when visiting their country of origin, compared to women. Condom use was higher with casual partners (42–65%) compared to steady partners (9–17%), but this leaves much room for improvement [33,37]. The results of a recent nation-wide Dutch study on sexual behaviour among young people under 25 years of age showed that 50 – 60% of TM and Surinamese young men reported consistent use of a condom, compared to approximately 30% of Dutch-Caribbean and Dutch boys [40]. The same study revealed that more TM boys have sexual experiences at a young age (12–17 years) and have more experience with anal sex and prostitutes than native Dutch boys [41]. In view of their early sexual start and because greater change can be expected at the beginning of sexual careers, it was decided to limit the intervention for TM men to the unmarried.

##### Analysis of behavioural determinants

The determinants of condom use were identified, and were categorized as personal, socio-cultural and physical environmental factors (Table 1), on the basis of the literature review and qualitative research. Similarities were seen in personal determinants between the AC and TM groups, but differences were apparent in socio-cultural environmental factors and physical environmental determinants.

**Table 1:** Identified important determinants of condom use

**Personal determinants**	**Socio-cultural determinants**	**Physical environmental determinants**
Awareness of importance of condoms in preventing pregnancies (AC, TM)*	Dominance of men in sexual relationship with women (AC, TM)	Price of condoms (AC, TM)
Awareness of importance of condoms in preventing STI (AC, TM)	Girls should marry as virgin (TM)	Easy access to condoms without social control of community (TM)
Self efficacy for condom use (AC, TM)	Unwanted pregnancy brings shame and problems with family (TM)	
Self efficacy for buying condoms (TM)	Sexual intercourse is not aloud outside marriage (TM)	
Self efficacy for carrying condoms for unexpected situations (AC, TM)	Multiple sexual relationships are more or less accepted (AC)	
Awareness of risks while having unprotected sex with new partner (AC, TM)	Approval of friends on condom use (AC, TM)	
Attitude towards condom use(AC, TM)	Approval of female partner on condom use (AC, TM)	
Use of oral contraceptives	Modelling behaviour of friends (AC, TM)	
Awareness of severity of STI and HIV (AC, TM)	Approval of roll models (older brothers and uncles) on condom use (TM)	
	Modelling behaviour of older brothers and uncles (TM)	

* AC: applicable to Afro-Caribbean group, TM: applicable to Turkish and Moroccan group

###### Personal factors

From the group discussions, the outcome expectancies or attitudes towards condoms as a means to prevent disease and pregnancy seemed rather positive. The literature reflects positive as well as negative attitudes towards condoms [42-46]. Negative beliefs, as described in the literature, were also expressed in the group discussions; condoms were reported to be associated with distrust and were not or less accepted within steady relationships or in marriage, while condoms were also considered to reduce pleasure [35,43-46].

Findings from the group discussions indicated that using oral contraceptives could be a barrier to using a condom, because there was no fear of an unwanted pregnancy.

"(....) You can simply look at a girl and see if you can trust her. How she acts and treats you. If she takes the pill it can happen that you will do it without (a condom). And I think that is just normal to do it without (a condom)." (AC group, 16–25 years)

In the group discussions, self-efficacy seemed important not only for using condoms, but also for buying them, as well as carrying them, so as to have condoms available at the right moment. The importance of self-efficacy for condom use is supported by the literature across different target groups [47,48].

###### Socio-cultural factors

The literature as well as the group discussions suggested that cultural aspects could not be ignored; for instance, machismo beliefs [43,49], the importance of female virginity, a taboo on premarital sex for the TM group, [43,45,50] and the association of condoms with prostitution.

In the TM group discussion, the majority was convinced that they would marry a virgin. A Turkish young man said that there is no need for a condom with the girl you are going to marry:

"*Because the woman you are marrying is always 100% virgin. So you are always the first one"(TM group, unmarried)*

Fear of an unwanted pregnancy seemed the biggest worry among the (unmarried) TM group in the group discussions. Social influence of peers for both groups, and of family and community for the TM men, seemed important in the group discussions, and was also described in literature [36,42,44,46]. The group discussions also indicated that norms and behaviours of the sex partner were important for both groups.

###### Physical environmental factors

It appeared from the group discussions that the lack of availability and accessibility and the price of condoms were barriers to their use.

Following is a citation illustrating the lack of preparation, and how a boy almost expects that the girl is the most responsible partner, which results in not having a condom available when it is needed:

"(...) If you are already involved in it, when you are just starting, and you know it will lead to it, that you are going to make love, then the girl has to say it. Usually it is the girl who says: do you have a condom? If I say no, and she says she hasn't one either. Then you are out of luck" (TM group, unmarried)

#### Step 2: Matrices of change objectives

Based on the needs assessment, the overall behavioural outcome was defined as "Using a condom in any sexual intercourse with new or casual partners in order to prevent STI and HIV". Next, POs were defined for both groups (Table 2). These POs address preparatory behaviour for condom use, namely deciding to use condoms, buying them, carrying them and then using them. The partner type specified in the POs differs between the two groups.

For the AC group, condoms appear to be especially accepted for casual sexual contacts in the prevention of HIV and STI; however, since use is still low, we have chosen to promote condoms in every casual (or new) sexual contact. In the (unmarried) TM group, premarital sex is almost by definition casual sex and condom use in every sexual contact should be recommended and encouraged. The fourth PO concerns the negotiating strategy on condom use. This negotiation strategy differs from that used in traditional condom use promotion interventions, which can be described as verbal and direct (negotiation with one's partner about safe sex). In this project we have chosen a non-verbal and direct strategy (simply take out a condom at the right moment) to increase condom use. The reason for this choice was based on the needs assessment that indicated that it was not common to discuss (un-) safe sex; indeed, discussing this topic seriously appeared to be a taboo. It seemed that a non-verbal and direct strategy was used by the men who reported most consistent condom use. Earlier studies also suggest that verbal, non-verbal, direct and indirect strategies are all effective in negotiating condom use and different strategies may be used depending on cultural or individual differences [51].

Next, important and changeable determinants of such negotiating behaviour had to be chosen. We used our own qualitative study, as well as a review of existing literature on safe sex interventions literature and reviews on correlates of condom use to determine which factors were both important to our target group and changeable. The personal determinants of self-efficacy, skills for buying, carrying and using condoms, awareness of peer behaviour, and subjective partner norms on condoms as well as the environmental determinant social norms regarding condoms were selected as most important determinants. Other important environmental determinants were the availability of condoms (the ease of obtaining and purchasing) and their accessibility (having a condom at hand). Socio-cultural determinants were considered not to be changeable in a short intervention, but were used to tailor the interventions culturally (some examples are given in the next section, which describes step 4 of IM). Attitude, risk perception and knowledge were considered important determinants, and would also be addressed. Subsequently, a matrix of change objectives was developed for each subgroup (Table 3).

#### Step 3: Select suitable theoretical methods and practical strategies

There is limited systematic evidence available on effective methods for prevention of STI and HIV in heterosexual men [5]. In a systematic review of Elwy et al [5] on interventions for heterosexual men for the prevention of HIV/STI, no single intervention could be identified as being more effective than others to change behaviour, increase knowledge or measure an intention to change. Methods in this review that are possibly applicable to the PO in our project are condom skill training, peer education, group discussions and individual counselling, focus on barriers to change, social aspects of condom use, video portrayal of acceptable normative condom behaviour, giving information and the provision of condoms. Furthermore, training with feedback has been found to be effective in promoting condom use [52-55].

Based on the review of the literature models or theories on behavioural change we used the following theories: Social Cognitive Theory [23], Implementation Intentions Theory [56], and inoculation theory [57]. Social cognitive theory was used to target self-efficacy, behavioural skills for condom use and preparative behaviour like carrying and negotiating condom use [18,52,54,55,58]. Methods for intervention were also derived from theory on implementation intentions for preparative behaviour like carrying condoms, plan to buy and store condoms [59], and from inoculation theory for countering negative arguments not to use condoms [18]. Moreover, the methods used provided information on risks and STI, persuasive arguments to use condoms [18] and anticipate a negative consequence after unsafe sex to change knowledge and outcome expectancies [16,55].

When translating the intervention map into practical strategies it was important to consider to whom and how the intervention was going to be delivered. The migrant health educators working with AC men did not consider organising group sessions of men to be realistic, since the recruitment of the focus groups for AC men for the needs assessment, and earlier MPHS experiences with groups sessions of these men [60] made clear that they were difficult to reach and recruit for group discussions. However, a successful approach that has been used repeatedly in this group is outreach work, where migrant health educators approach AC men at different locations, such as at festivals, on streets, or at sport tournaments to give information on safe sex accompanied by supplying free condoms. We decided to link our intervention to these ongoing activities and provide the health educator with a new tool. As a consequence of the format of the intervention, the number of change objectives for the AC group had to be reduced, because only a limited number of determinants (attitude and self-efficacy) could be discussed during outreach work.

The experience with recruiting the TM men for group discussions was much better. Through organisations in neighbourhoods, youth groups and Islamic organisations, groups could be set up quite easily. Therefore, for this target group, a small group intervention was designed in which the selected methods (e.g. discussion in groups on norms and attitudes, training of skills with feedback, modelling of skills, video films on attitudes and norms) could be included. Examples of methods and strategies are presented in Table 4.

**Table 4:** Methods for determinants, translated in intervention components for Turkish/Moroccan group (selected examples)

Determinant	Change objectives	method/strategy	intervention components
Attitude	Man acknowledges advantages of condom use as a protection against STI	modelling, using new positive arguments, self re-evaluation, persuasion, group discussion	Discussion in response to posters picturing Moroccan and Turkish young men giving statements about safe sex Game "Save your head": issue with discussion on non-health issues as advantage of using condoms.
Risk perception	Man explains that from appearances you can't tell if someone is infected with HIV/STI.	provide basic factual information, personalising risk, consciousness raising.	Game "Save your head": issue with discussion on unsafe sex with girls who you think are a virgin. Information is given on risks of different sexual techniques. Game "Save your head": issue with discussion on the question "with which women do you not need to use a condom?" Explaining misconceptions.
Self efficacy	Man expresses confidence that he will use condoms, also in a situation that a woman does not request a condom.	anticipating on difficult situations modelling, active participation.	Assignment: "Plan your sex", interviewing each other on preparative behaviour and making plans and how to be prepared for safe sex. Assignment preceded by video with modelling of behaviour.
Self efficacy	Man describes where he puts away his condoms, to have them available always.	anticipating on difficult situations modelling, active participation.	Assignment: "Plan your sex", interviewing each other on preparative behaviour and making plans and how to be prepared for safe sex.
Self efficacy	Man expresses confidence that he will have condoms available always.	modelling, making action plans, forming implementation intentions, identification of barriers	Video with role models showing that they are able to solve problems with the availability of condoms in planned and unplanned sex. Assignment: "Plan your sex", interviewing each other on preparative behaviour and making plans and how to be prepared for safe sex.
Self efficacy	Man describes a plan how he carries condoms always	making action plans forming implementation intentions identification of barriers	Assignment: "Plan your sex", interviewing each other on preparative behaviour and making plans and how to be prepared for safe sex. Assignment on buying condoms
Subjective partner norm	Man knows opinion of most women that they also want to use a condom in a new or casual sexual contact.	Modelling group discussion	Discussion in response to posters picturing Moroccan and Turkish young men giving statements about safe sex. Game "Save your head" : issue with discussion on the matter

To boost personal availability of condoms in both groups we decided to develop a holder for one or more condoms and offer it to the men free of charge. After consulting young men in the additional focus group, a trendy shoulder bag and wristband were chosen.

#### Step 4: Design a programme plan

We designed a small group intervention in two sessions for the TM unmarried men. Table 4 illustrates how change objectives were translated into intervention components for the TM group. For example, modelling was used to stimulate discussion within the group. For this purpose, twelve posters were made picturing TM young men making statements about safe sex. For example, on (new) beliefs, resisting (supposed) female pressure, and addressing cultural norms on premarital sex. To make the interventions culturally appropriate, the pictures with young men making statements about safe sex comprise some statements that refer to the taboo on premarital sex and negative anticipated reactions of the family if an STI or unwanted pregnancy occurs as a consequence of unsafe sex (for example: "My family does not need to know I use condoms, but the girl has to!" and "I don't like problems, I use a condom"). Furthermore, a game ("Never Lose Face") was designed for discussing risk perception, partner influence and personal behaviour, as well as addressing (presumed) differences in male/female beliefs and norms on sexuality and the purchasing of condoms. Most components of the group intervention were tested during May and June 2005. Forty young men aged between 15 and 19 attended four two-hour group sessions. It took more time than planned to organise the groups for the intervention, as a consequence only one session could be held for each group instead of the two sessions that were scheduled. The exercise for forming implementation intentions ("plan your sex") and the assignment for buying condoms were therefore omitted (see Table 4). The assignment for buying condoms was skipped because this topic was also incorporated as an assignment in the game "Never Lose Face". The exercise ("plan your sex") included a video or DVD fragment; recorders to play the tape were not always available at the sites. Furthermore, the young men did not want to choose a partner and interview each other in pairs, as was the purpose of the assignment. They preferred to speak in the group instead of one-to-one, so this assignment was carried out as a group discussion. Because this item could not be carried out as planned and because of time restrictions, it was omitted in the following groups. The sessions were highly appreciated by the young men attending and were judged positively on personal relevance.

For the AC group, a set of playing cards was designed ("put your cards on the table"). The prevention officer, who was very experienced in working with the AC target group, sketched the situations and tools to bring to a date. To match the learning objectives, some revisions were made in collaboration with the researcher. For example, adding a playing card with the subject alcohol is a means to discuss the influence of alcohol on unsafe sexual behaviour. Then, a professional designer made a first prototype of the playing cards. These were tested among AC men during regular health education activities. The images of the cards needed some revision, a picture of a sexy women was put on the back of the playing cards, and some of the situations were described somewhat less explicitly (e.g. the statement that he is anticipating that he is having sex with a women at her place was revised to she asks him to go home with her). The men also wanted the real life situations described more explicitly; for example, it needed to be stated clearly that in a certain situation the man was single, or the man had two women. Also the colours of the cards were changed.

In the playing cards the situations were adapted to common practices in relationships of Afro-Caribbean culture. For example, one of the situations is about a man who lives together with one women, but also has sexual relations with another. A health educator can play the game with individual men at outreach locations. The cards represent real life situations (e.g. meeting a woman in a bar) that may lead to sexual encounters. By choosing additional cards the man is asked about his behaviour and choices regarding preparing and pursuing safe sex (for example, what would the men bring to a date). During the game attitude aspects, risks in unsafe sex and preparation behaviour are discussed and condom skills are practiced using a dildo. The intervention was tested among 72 men in August 2005. On different locations where the MPHS health educators work, they played the card game with men from the appropriate target groups. These outreach locations were an urban festival, a soccer tournament, meeting spots in the city centre and in a Cape Verdean church. A small questionnaire was handed out before and after the game for evaluation. The game of cards was received very well: the men could identify themselves very well in the situations and the game led to discussions on safe sex.

## Discussion

Our study is one of the few focusing on the systematic development of HIV/STI prevention interventions for heterosexual migrant men. The needs assessment showed the importance of personal factors such as self-efficacy and attitude as well as socio-cultural factors and the accessibility and availability of condoms. Two interventions, one suitable for outreach work and the other for group sessions, were developed and pre-tested among AC and TM men. The project also serves as an example for development in the daily practice of public health of evidence and theory led interventions. It has the advantage of being carried out within the established framework of collaboration between a municipal health service and a university department with expertise on planned theory and evidence-based development of health promotion interventions.

We experienced that using IM in designing interventions in practice requires flexible handling of a more compressed IM protocol than that described by the protocol developers, since the application according to the IM textbook took more time than was available. This problem with application of IM according to textbook instructions is also described by other authors [61,62]. Although IM is a valuable checklist and guide to take the right steps in developing an intervention, it does include the risk of remaining in a lengthy endless process of doing further research on determinants, developing matrices for more and more specified performance objectives, and moving back and forth between intervention development, pre-testing and further exploratory research. The time and resources to do this are not available in the day-to-day practice of a municipal public health service, and applying IM according to the full instructions is therefore difficult in the practice of local and regional public health promotion.

Furthermore, it appears that there are too few examples in the literature on culturally grounded HIV prevention and that the examples that are available are only minimally described [63]. This paper on the development of HIV prevention interventions for two different ethnic groups will therefore add to the development of practice for the design of culturally grounded interventions.

The validity of the needs assessment can be considered a limitation of this study. Literature findings on the target population were scarce. The group discussions were held among a selective group of men, non-attendance was high in the AC group, and not all groups were sufficiently filled. However, research on focus group discussions among these target groups often fails, and despite these difficulties, we believe we have contributed to further exploration of the evidence on behavioural determinants on safe sex among migrant men in the Netherlands, a group that is difficult to reach with standard prevention efforts. The reality of prevention work within the MPHS forced decisions on the feasibility of influencing the most important behavioural determinants. The consequence of the initial choice of self-efficacy and social influence as the most important target determinants for the intervention meant that more intensive and expensive interventions were needed in comparison to traditional prevention. For example, a more intensive approach needs to be used for the AC men, such as a theatre play performed on popular festivals with AC audience to portray positive norms on safe sex, a popular rapper rapping on negative and positive outcome expectancies of unsafe sex, and training peers/opinion leaders as condom experts. However, this was not possible within the available time, budget and competence of the MPHS migrant prevention workers and health educators. Consequently, the AC intervention was limited to personal determinants; self-efficacy was only briefly addressed and physical environmental factors were not addressed in the interventions.

Our experience has taught us that a team of prevention officers and researcher is of great value and the collaboration between health workers from the MPHS and researchers from the university department was most helpful to combine setting-specific practical experiences with theoretical insights of more general validity. The project is practice-driven; a prevention officer working in the MPHS was responsible for the original plan for developing interventions for migrant men with the guidance of the IM protocol. University-based experts got involved with further development of the plan. During the development phase, the prevention officers and researcher worked together closely. Because of their experience with health education among the target groups, the prevention officers were best suited to suggest the further details of the intervention so that it would fit in with their routine of prevention work and the preferences of the target groups. The researcher guaranteed that the plans were in accordance with the theoretical strategies and fitted within the change objectives matrices. The main tasks of the health policy adviser, who was also experienced in IM, were to make sure that the targets of the subsequent steps were reached, and to organize and manage the consultations with the experts and brainstorm sessions. If our stepwise IM-inspired development process has made one thing clear, it is that little is known about (safe) sexual behaviour of heterosexual migrant men and its determinants. To develop better evidence-based interventions, more research in this field is needed. A formal evaluation of the interventions proposed in the present paper can contribute to this goal.

## Conclusion

This project has shown that it is feasible to apply IM in the daily practice of the MPHS for the development of HIV/AIDS prevention interventions. However, working according to IM requires more time than is usually available in public health service and sufficient theoretical knowledge and experience with technical IM aspects has to be available.

## Competing interests

The author(s) declare that they have no competing interests.

## Authors' contributions

MW carried out the study, participated in the design of the study, performed the qualitative analyses and drafted the manuscript. KvdH carried out the study, participated in the design of the study and helped to draft the manuscript. JB discussed interpretation of results and helped draft the manuscript. OdZ participated in the design of the study, helped draft the manuscript and coordinated the study. All authors read and approved of the final manuscript.

## Pre-publication history

The pre-publication history for this paper can be accessed here:


